# Date Palm Waste Compost Application Increases Soil Microbial Community Diversity in a Cropping Barley (*Hordeum vulgare* L.) Field

**DOI:** 10.3390/biology12040546

**Published:** 2023-04-03

**Authors:** Emna Ghouili, Ghassen Abid, Richard Hogue, Thomas Jeanne, Joël D’Astous-Pagé, Khaled Sassi, Yassine Hidri, Hatem Cheikh M’Hamed, Anil Somenahally, Qingwu Xue, Moez Jebara, Rim Nefissi Ouertani, Jouhaina Riahi, Ana Caroline de Oliveira, Yordan Muhovski

**Affiliations:** 1 Laboratory of Legumes and Sustainable Agrosystems, Centre of Biotechnology of Borj-Cedria, (L2AD, CBBC), Hammam-Lif 2050, PB 901, Tunisia; 2 Microbial Ecology Laboratory, Research and Development Institute for the Agri-Environment (IRDA), Einstein Street 2700, Québec City, QC G1P 3W8, Canada; 3 Laboratory of Agronomy, National Agronomy Institute of Tunisia (INAT), University of Carthage, Avenue Charles Nicolle, Tunis-Mahrajène 1082, BP 43, Tunisia; 4 Olive Tree Institute, Laboratory of Integrated Olive Production in the Humid, Sub-humid and Semi-arid Region (LR16IO3), Cité Mahragène 1082, BP 208, Tunisia; 5 Agronomy Laboratory, National Institute of Agronomic Research of Tunis (INRAT), University of Carthage, Hedi Karray Street, Ariana 2049, Tunisia; 6 Department of Soil and Crop Sciences, Texas A&M University, 370 Olsen Blvd., College Station, TX 77843-2474, USA; 7 Texas A&M AgriLife Research and Extension Center, Amarillo, TX 79106, USA; 8 Laboratory of Plant Molecular Physiology, Centre of Biotechnology of Borj Cedria, Hammam-Lif 2050, BP 901, Tunisia; 9 Technical Center for Organic Agriculture, Chott Mariem, Sousse 4042, BP 54, Tunisia; 10 Biological Engineering Unit, Department of Life Sciences, Walloon Agricultural Research Centre, Chaussée de Charleroi, 5030 Gembloux, 234 BP, Belgium

**Keywords:** barley, date palm waste compost, high-throughput sequencing, microbial community, quantitative real-time PCR (qPCR)

## Abstract

**Simple Summary:**

The objective of this study was to determine the effects of compost application on soil bacterial and fungal communities at the tillering, booting and ripening stages of barley plant growth. The two treatments were unfertilized (control) and compost treatment (30 t ha^−1^). The high-throughput sequencing approach and real-time quantitative PCR method were performed to study the characteristics of soil bacterial and fungal communities. Compost addition significantly altered the compositions of bacterial and fungal communities. Our results revealed that the Chao1 index of the fungal community increased at the tillering and booting stage, while that of the bacterial community decreased at the tillering and ripening stage. Moreover, compost decreased the Shannon index of fungal and bacterial communities, especially at the tillering stage. The dominant bacterial phyla found in the samples were *Proteobacteria* and *Actinobacteria* while the dominant fungal phyla were *Ascomycota* and *Mortierellomycota*. Identified bacteria were mainly involved in energy metabolism, amino acid metabolism, metabolism of cofactors and vitamins and carbohydrate metabolism while fungi were saprotroph, pathotroph symbiotroph and endophyte. Overall, date palm waste compost addition could be considered as a sustainable practice for establishing a healthy soil microbiome and subsequently improving the soil quality and barley crop production.

**Abstract:**

Application of date palm waste compost is quite beneficial in improving soil properties and crop growth. However, the effect of its application on soil microbial communities is less understood. High-throughput sequencing and quantitative real-time PCR (qPCR) were used to evaluate the effect of compost application on the soil microbial composition in a barley field during the tillering, booting and ripening stages. The results showed that compost treatment had the highest bacterial and fungal abundance, and its application significantly altered the richness (Chao1 index) and α-diversity (Shannon index) of fungal and bacterial communities. The dominant bacterial phyla found in the samples were *Proteobacteria* and *Actinobacteria* while the dominant fungal orders were *Ascomycota* and *Mortierellomycota*. Interestingly, compost enriched the relative abundance of beneficial microorganisms such as *Chaetomium*, *Actinobacteriota*, *Talaromyces* and *Mortierella* and reduced those of harmful microorganisms such as *Alternaria*, *Aspergillus* and *Neocosmospora*. Functional prediction based on Phylogenetic Investigation of Communities by Reconstruction of Unobserved States (PICRUSt) showed that amplicon sequence variant (ASV) sequences related to energy metabolism, amino acid metabolism and carbohydrate metabolism were associated with compost-treated soil. Based on Fungi Functional Guild (FUNGuild), identified fungi community metabolic functions such as wood saprotroph, pathotroph, symbiotroph and endophyte were associated with compost-treated soil. Overall, compost addition could be considered as a sustainable practice for establishing a healthy soil microbiome and subsequently improving the soil quality and barley crop production.

## 1. Introduction

Hot and dry Mediterranean agroecosystems including Tunisia are characterized by low soil fertility and stressful conditions that limit crop production. Organic fertilizers are carbon-based compounds that promote growth, productivity and quality of plants. Among organic fertilizers, compost contains a significant proportion of plant nutrients. Compost application can increase soil organic matter, soil nutrient availability, water use efficiency and soil properties, resulting in increased crop yields [1]. Interestingly, organic fertilizers including compost are not only a source of organic matter and nutrients, but also stimulate the microbial population and boost physicochemical and biological properties of the soil and, thereby, sustainable agricultural production [2]. Several studies have reported that the application of compost increases microbial biomass and activities and nutrient uptake by the plants and decreases disease incidence, which promotes the plant growth [3]. Date palm waste compost showed significantly improved plant growth and barley grain yield [4]. This may be associated with the increase in macronutrient uptake due to the induction of several genes involved in uptake and transport of nitrate, ammonium and phosphorus in the leaves and roots of barley plants [4].

It is well known that soil microorganisms interact with the roots of plants and play an important biological role in plant growth and health. These beneficial microorganisms, including arbuscular mycorrhizal fungi (AMF), nitrogen-fixing symbiotic bacteria and plant-growth-promoting rhizobacteria (PGPR), can increase the supply of mineral nutrients to the plant, control phytopathogens by preventing the growth or activity of pathogens and staminate plant growth by production of growth regulators such as phytohormones [5]. In this context, soil microbial communities strongly influence plant traits and functions. Metabolic analysis revealed that changes in belowground bacterial communities significantly influenced pathways involved in grapevine (*Vitis vinifera* L.) plant defense and production of fruit secondary metabolites [6]. Moreover, reduction in the complexity of rhizosphere soil microbial communities reduced chlorophyll content, flower numbers and size of cabbage (*Brassica rapa* L.) plants [7]. Similar results were reported in *Ipomoea purpurea* plants [8]. Several studies reported that application of compost into soil significantly affected soil microbiome structure and function in different agroecosystems [9,10,11]. A short-term (227 days) fertilization study showed that compost application increased radish plants biomass, modified the composition of the soil microbial populations, altered network topography and changed network hub taxa [3]. On the other hand, a long-term (25 years) fertilization study revealed that application of compost in the winter wheat–corn rotation system greatly increased levels of beneficial microbes and reduced the level of harmful [12]. 

In Tunisian oasis ecosystems, the application of organic fertilizers such as date palm waste compost is strongly recommended in order to improve the principal soil physicochemical properties (organic matter and water retention capacity) which can positively affect the plant yield and promote agroecosystem health [4,13,14]. However, the effect of date palm waste compost application on the structure and diversity of the rhizosphere bacterial community has not been elucidated and is less understood. Therefore, MiSeq amplicon sequencing of bacterial 16S rRNA gene and fungal ITS rDNA region data were used to investigate the effects of date palm waste compost on soil microbial diversity, composition and community structure under a 2-year fertilization study in the field during the tillering, booting and ripening stages of the barley (*Hordeum vulgare* L.) growth cycle.

## 2. Materials and Methods

### 2.1. Experimental Site and Design

The experiments related to the study were carried out in an open field of NGmOASOC (Association for Saving Oasis of Chenini, Gabes, Tunisia) during the 2020 growing season. The annual average temperature and precipitation were 21 °C and 170 mm, respectively, suggesting a Mediterranean arid climate. Physicochemical properties of the experimental field are given in Table 1.

Treatments were arranged in randomized block designs with three replications and a total of 6 experimental plots. Each plot covered 10 m^2^ (2.5 m wide by 4 m long) and the space between them was 1 m. The experimental treatments were: (T1) unamended soil and (T2) soil amended with 30 t ha^−1^ of compost and mixed in the upper 15–20 cm soil one day before seeding. Date palm waste compost obtained by mixing cow manure and date palm leaves (50:50) was provided by composting station of ASOC and its physical, chemical and biological properties are shown in Table 2. The barley seeds were sown on 16 December in 2020 growing season using seed rate of 120 kg ha^−1^ employing Sahli cultivar.

### 2.2. Soil Sampling

Soil samples were collected at tillering (S1), booting (S2) and ripening (S3) stages. In each plot, six soil core samples (0–20 cm depth) were collected and mixed to prepare a composite sample. Immediately after sampling, visible root fragments and stones were manually removed and the soil samples were placed in sterile plastic bags, sealed and then were preserved in ice packs and taken to the laboratory. An aliquot of the soil samples was stored at −80 °C until DNA extraction and subsequent molecular analysis and the rest was air-dried for physicochemical analyses.

### 2.3. DNA Extraction, Amplicon Library Preparation and Sequencing

Soil DNA was isolated from 0.5 g of soil samples using the FastDNA™ SPIN Kit for Soil (MP Biomedicals, Solon, OH, USA), following the manufacturer’s instructions. The quality and quantity of the extracted soil DNA were evaluated by UV spectrophotometry (Biophotometer, Eppendorf, Mississauga, ON, Canada). Measurements were performed at wavelengths of 260, 280, 230 and 320 nm.

For bacterial community analysis, the V4 hypervariable region of 16S rRNA gene was amplified by polymerase chain reaction (PCR) using the primers set [15], 515FB: 5′-GTGCCAGCMGCCGCGGTAA and 806 RB: 5′-GGACTACHVGGGTWTCTAAT. For fungal community analysis, the fragments of ITS1 rDNA region were amplified by PCR using the primers set [16], BITS: 5′-CTTGGTCATTTAGAGGAAGTAA and B58S3: 5′-GAGATCCRTTGYTRAAAGTT. Sequencing was performed by Illumina MiSeq instrument at the Genomic Analysis Platform (IBIS, Laval University’s, Quebec City, QC, Canada).

The total PCR reaction volume was 25 μL and containing 5 µL of template DNA, 5 µL Q5 buffer (NEB, Whitby, ON, Canada), 200 mM of each dNTP, 0.25 mM of each primer and 0.5 U of Q5 High-Fidelity DNA polymerase (NEB, Whitby, ON, Canada). The amplification reaction was performed as follows: 30 s at 98 °C, 35 cycles of 10 s at 98 °C, 10 s at 55 °C for prokaryotes and 50 °C for fungi, 30 s at 72 °C with a final extension for 2 min at 72 °C. The PCR products were purified with Axygen PCR cleanup kit (Fisher Scientific, Nepean, ON, Canada). The quantity of the purified PCR products was assessed using an Epoch microplate reader (Agilent, Santa Clara, CA, USA) and the 260/280 ratio was used to detect the quality of the PCR samples. In the second PCR step, 50- to 100-fold serial dilutions of this purified product, barcodes (dual-indexed) and Illumina adapters were used under identical cycling parameters to the first PCR [17]. However, only 12 cycles were performed to reduce complexity of amplicon libraries. Gene libraries were then combined in equimolar concentrations and sequenced on the Illumina MiSeq platform using a 2 × 300 bp sequencing kit.

### 2.4. Quantitative Real-Time PCR (qPCR) Analysis

qPCR was performed to determine the abundances of total bacteria and fungi in the bulk soil, according to previously described protocol [18]. Eub338: 5′-ACTCCTACG GGAGGCAGC AG and Eub518: 5′-ATTACC GCGGCTGCTGG [19] and FF390: 5′-CGATAACGAACGAGACCT and FR1: 5′-AICCATTCA ATCGGTAIT primers [20] were used for prokaryotes and fungi, respectively. Amplifications were carried out using a CFX96 Touch Real-Time Detection (Biorad, Hercules, CA, USA) and SYBR green qPCR mix (Qiagen, Toronto, ON, Canada). The qPCR assays were carried out as follows: initial preheating at 95 °C for 15 min, followed by 40 cycles: 95 °C for 1 min, annealing temperature (53 °C for bacteria and 51 °C for fungi) for 30 s followed by 72 °C for 1 min. Standard curves were generated using 10-fold serial dilutions of M13 (pBluescript II). Gene copy numbers were normalized per gram of dry soil. Each assay was performed in triplicate and the mean values were compared using Duncan’s test when a significant F-value was obtained (*p* < 0.05).

### 2.5. Data Analysis and Statistics

Bacterial 16S rRNA gene and fungal ITS rDNA region sequencing data were analyzed using QIIME2 next-generation microbiome bioinformatics pipeline for comparative metagenomics study [21]. Amplicon sequence variants (ASVs) were determined by the Divisive Amplicon Denoising Algorithm 2 (DADA2) method [22]. Here, the ASV nomenclature replaces the well-known operational taxonomic unit (OTU) appellation [23]. *Cutadapt* tool was used to find and remove adapter sequences from high-throughput sequencing reads. To assess the differences between prokaryotes and fungi, weighted UniFrac distances were calculated based on the phylogenetic differences. A principal coordinate analysis (PCoA) was conducted on a UniFrac distance matrix using vegan package on R. A one-way permutational multivariate analysis of variance (PERMANOVA) was performed based on UniFrac responses to determine whether bacterial and fungal communities were significantly influenced by date palm waste compost treatment, with 999 permutations and beta-group-significance command in QIIME2 [24]. A multivariate pairwise test for pairwise comparisons was also performed to estimate the variance across all paired groups. Nonparametric test results are reported in this study; Kruskal–Wallis test followed by Wilcoxon test were used for pairwise comparisons between groups using *stat_compare_means* function of the *ggpubr* package version 0.4.0. in R version 4.1.1. Three alpha-diversity indices reflecting the microorganism community richness (Chao1) [25] and the microorganism community *alpha*-diversity (Shannon and Simpson) [26] were calculated using QIIME2 program. The relative abundance for the phylum and order levels were represented as stacked bar plots using phyloseq version 1.41.1 [27] and genus levels were visualized as a heatmap using the packet ampvis2 version 2.7.32 [28]. A differential DESeq2 analysis was performed to compare the abundances of each ASV using the DESeq2 packet version 1.38.1 [29]. The bacterial functional potential of the soil was estimated using the PICRUSt2 tool for predicting functional abundances [30]. The Kyoto Encyclopedia of Genes and Genomes (KEGG, https://www.genome.jp/kegg/ accessed on 10 June 2022) module database was completed in July 2022 [31]. To estimate the fungal functional potential of the soil, the FUNGuild tool (http://www.funguild.org/ accessed on 10 June 2022) was used [32].

## 3. Results

### 3.1. Changes in the Soil’s Bacteria and Fungi Abundances

**Figure 1:**
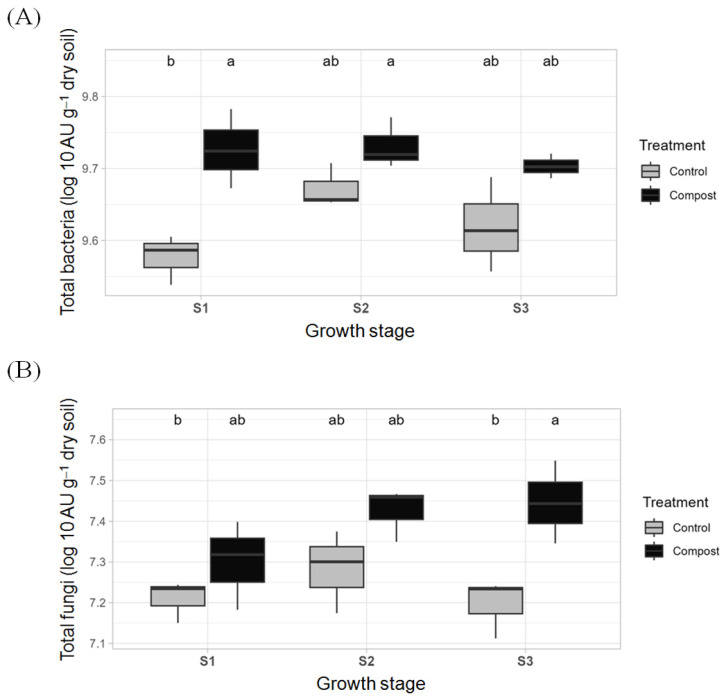
Quantitative PCR (qPCR) measurements of bacterial abundances (**A**) and fungal abundances (**B**) in the soils that grew barley without or with date palm waste compost at tillering (S1), booting (S2) and ripening stage (S3). Different letters above columns indicate significant differences (*p* < 0.05, Duncan’s test).

Total bacteria and fungi population using the 16S rRNA gene and ITS rDNA region, respectively, was investigated to determine the effect of compost application on the soil microbiome community. Quantitative PCR (qPCR) results showed that in comparison to control soils, the compost treatment significantly increased bacterial abundance in soil sampled at S1 and to a lesser extent at S2, while fungal abundance increased at S3 and to a lesser extent at S1 stages (Figure 1). Overall, total bacteria abundance (Figure 1A) was higher than fungi abundance by more than two log values (Figure 1B) in all barley growth stages in compost-treated and untreated soils. Moreover, adding compost increased the fungi-to-bacteria ratio at each growth stage of barley.

### 3.2. Effect of Compost Application on Bacterial and Fungal Diversity in Soil

**Figure 2:**
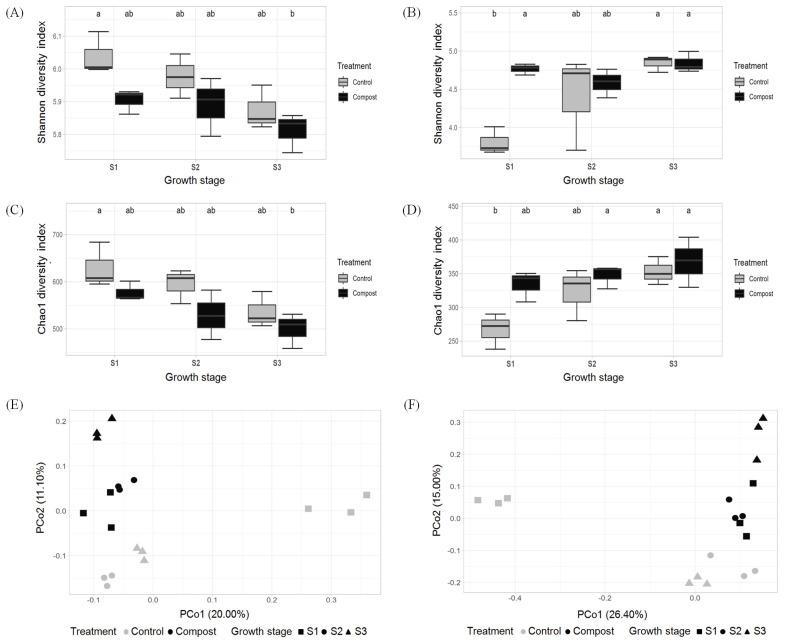
Microbial diversity analysis. Box plots represent *alpha*-diversity indices, including Shannon index of bacterial (**A**) and fungal communities (**B**); Chao1 index of bacterial (**C**) and fungal communities (**D**) and *beta*-diversity analysis of principal coordinates (PCoA) of bacterial (**E**) and fungal communities (**F**).

Genetic diversity of bacterial and fungal communities was evaluated using high-throughput sequencing of the 16S rRNA gene and ITS rDNA region. Shannon and Chao1 diversity indices as well as principal coordinate analysis (PCoA) were determined to measure soil sample microbial diversity in communities during the S1, S2 and S3 growth stage separately (Figure 2). Overall, in bacterial communities, Shannon and Chao1 diversity indices showed higher mean values than fungal communities.

Shannon diversity indices of the bacterial community in untreated soil were slightly higher than those measured in compost-treated soils for all barley growth stages (Figure 2A). However, the Shannon diversity indices of the fungal community were higher in compost-treated soils solely during the S1 growing period (Figure 2B). The Chao1 diversity index of bacterial communities showed slightly higher values in the control compared to the compost treatment during the S1 and S3 growth stages of the barley plant (Figure 2C). In the fungal community, the Chao1 values were slightly higher in compost-treated soils during the S1 and S2 periods (Figure 2D). Interestingly, alpha-diversity analysis showed that in the bacterial community, Shannon and Chao1 mean values decreased, while in the fungal community, they increased progressively during the growing season in control and compost-treated soils, except the Shannon index values of the fungi community in compost-treated soils.

The principal coordinate analysis (PCoA) based on UniFrac distances revealed that the beta-diversity of soil (0–20 cm depth) bacterial (Figure 2E) and fungal (Figure 2F) communities clustered separately at different barley growth stages. Interestingly, the soil bacterial or fungal communities of the control treatment at the S1 period were grouped separately from those of the control soils at the S2 and S3 periods and from those of compost-amended soils at any given barley growth periods (Figure 2E,F). For the bacterial community, the PCoA1 and PCoA2 axes explained, respectively, 20.00% and 11.10% of the total variation, while for the fungal community they explained 26.40% and 15.00% of the total variation (Figure 2E,F).

### 3.3. Taxonomic Changes to Bacterial and Fungal Populations in Soil

**Figure 3:**
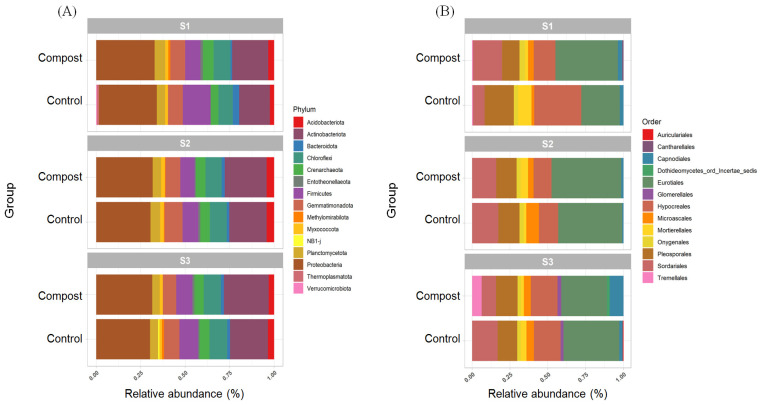
Relative abundance of bacterial phyla (**A**) and fungal orders (**B**) detected in soil after date palm waste compost treatment at tillering (S1), booting (S2) and ripening stage (S3).

In all stages of barley growth, significant differences were observed in the relative abundance of bacterial phyla and fungal orders after applying compost (Figure 3). Among the 15 phyla, *NB1-j* was not identified, whereas the *Proteobacteria*, *Firmicutes* and *Actinobacteriota* were the three top bacterial phyla in the control and compost treatment during the S1, S2 and S3 barley growth stage (Figure 3A). Application of compost significantly affected the relative abundance of bacterial phyla at the tillering (S1) stage of barley growth. Indeed, during S1, *Actinobacteria* was second only to *Proteobacteria* in dominance. Of these, the phyla *Entotheonellaeota* and *Methylomirabilota* were observed in the compost treatment while *Thermoplasmatota* and *Verrucomicrobiota* occurred only in the control treatment. Moreover, compost application increased the relative abundance of *Actinobacteriota*, *Acidobacteriota*, *Chloroflexi*, *Crenarchaeota* and *Planctomycetota*; however, it decreased the abundance of *Firmicutes* and *Bacteroidota*. At the booting stage (S2), *NB1-j*, *Thermoplasmatota* and *Verrucomicrobiota* phyla were not identified in control and compost samples. The relative abundance of *Proteobacteria* and *Actinobacteriota* increased, while *Planctomycetota* and *Gemmatimonadota* decreased in the compost treatment compared with control. Surprisingly, *NB1-j* occurred in only the control treatment, while *Thermoplasmatota* and *Verrucomicrobiota* were not identified in control and compost samples during the ripening stage (S3). The application of compost mainly increased the abundance of *Proteobacteria* and *Actinobacteriota* but decreased the abundance of *Firmicutes*, *Gemmatimonadota*, *Methylomirabilota*, *Myxococcota* and *NB1-j*.

For fungal communities, a total of 13 orders were identified. The phyla *Eurotiales*, *Hypocreales*, *Sordariales* and *Pleosporales* were dominant (Figure 3B). Significant differences were observed in the relative abundance of fungal orders after applying date palm waste compost at all barley growth stages. Compost treatment significantly increased the abundance of *Eurotiales*, *Sordariales*, *Microascales*, *Onygenales* and *Capnodiales*; however, it significantly decreased the abundance of *Hypocreales*, *Pleosporales* and *Mortierellales* at the tillering (S1) stage of barley growth. Moreover, *Cantharellales* occurred in only the compost treatment, while it was not identified in the control. At the booting stage (S2), a large proportion of the fungal community consisted of *Eurotiales* in the control and compost treatments. Of eight identified orders, compost treatment increased the abundance of *Eurotiales*, *Mortierellales* and *Capnodiales*; however, it decreased the abundance of *Microascales*, *Hypocreales*, *Onygenales*, *Sordariales* and *Pleosporales*. The diversity and the relative abundance in the fungal communities changed at the ripening stage (S3) compared to S1 and S2. Indeed, 12 fungal orders were detected. *Auriculariales* were identified in control but not in compost samples during the ripening stage (S3). In contrast to the S1 and S2 growth stage, compost treatment significantly decreased the abundance of *Eurotiales*. Moreover, *Microascales*, *Onygenales*, *Mortierellales* and *Sordariales* showed a decrease in the compost treatment compared to control. Interestingly, *Glomerellales* and *Dothideomycetes_ord_Incertae_sedis* occurred in only S3. In this context, *Glomerellales* appeared in the control and compost treatment, while *Dothideomycetes_ord_Incertae_sedis* and *Tremellales* appeared only in the compost treatment. Among the 12 most abundant fungal orders, compost treatment increased the relative abundance of *Capnodiales*, *Glomerellales*, *Hypocreales* and *Pleosporales*.

**Figure 4:**
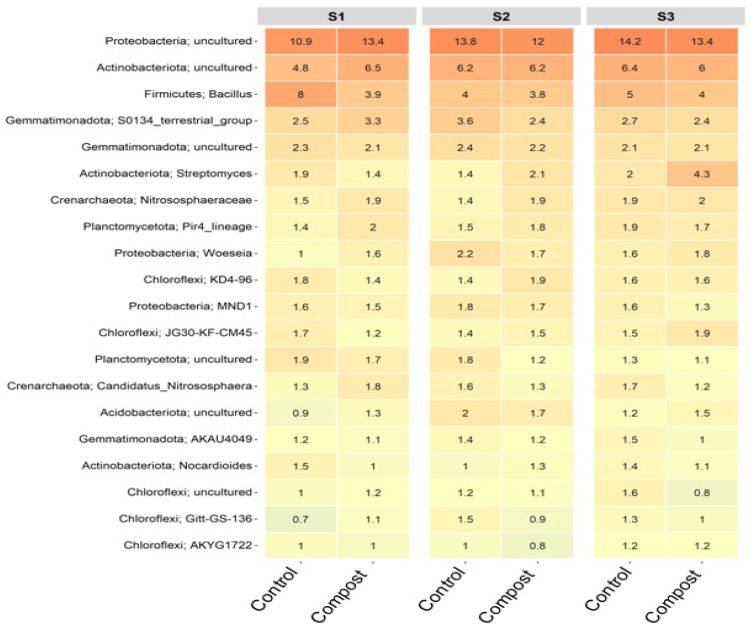
The heatmap of the correlation between bacterial phyla and treatments at tillering (S1), booting (S2) and ripening stage (S3).

**Figure 5:**
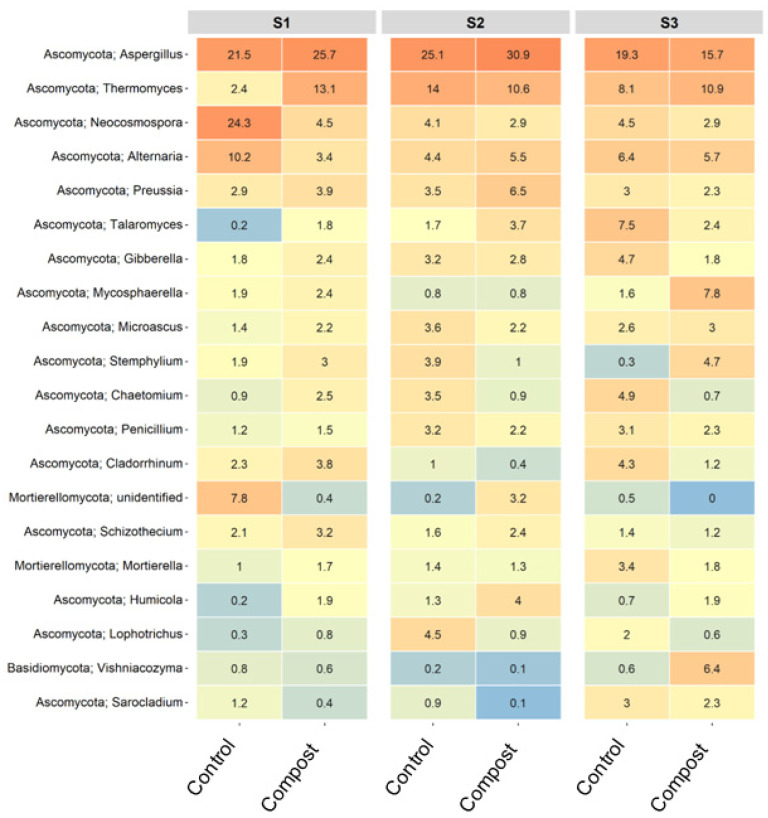
The heatmap of the correlation between fungal orders and treatments at tillering (S1), booting (S2) and ripening stage (S3).

A heatmap was used to further explore the relationships among the treatments and the microbial community structure in the barley-cultivated soil. In this context, the top 20 most abundant bacterial (Figure 4) and fungal phyla (Figure 5) were analyzed. As can be seen from Figure 4, among the bacteria, *Firmicutes*, *Actinobacteriota* and *Proteobacteria* had the highest abundances in the control and compost treatment at all studied barley growth stages.

Similarly, *Ascomycota,* followed by *Mortierellomycota* and *Basidiomycota,* were identified in fungal phyla (Figure 5). Moreover, as shown in Figure 4 and Figure 5, the abundance of some bacterial and fungal phyla also varied among the different treatment and between barley growth stages. For example, *Firmicutes* had its highest abundance in the control treatment and the lowest abundance under compost treatment (Figure 4). The abundance of *Actinobacteriota* was highest only under compost treatment during the S3 stage compared to other samples.

As shown in Figure 5, the abundance of *Ascomycota* (*Neocosmospora*, *Alternaria*) and *Mortierellomycota* (Unidentified) were highest only in the control treatment at S1 but decreased in other samples. In contrast, the abundance of *Ascomycota* (*Talaromyces*, *Humicola* and *Lophotrichus*) were lowest only in the control treatment at S1 but increased in other samples. Taken together, heatmap data showed that the compost treatment and the barley growth stage influenced the abundance of the first 20 most abundant soil microorganisms.

### 3.4. Major ASVs within Barley Soil Microbial Communities

**Figure 6:**
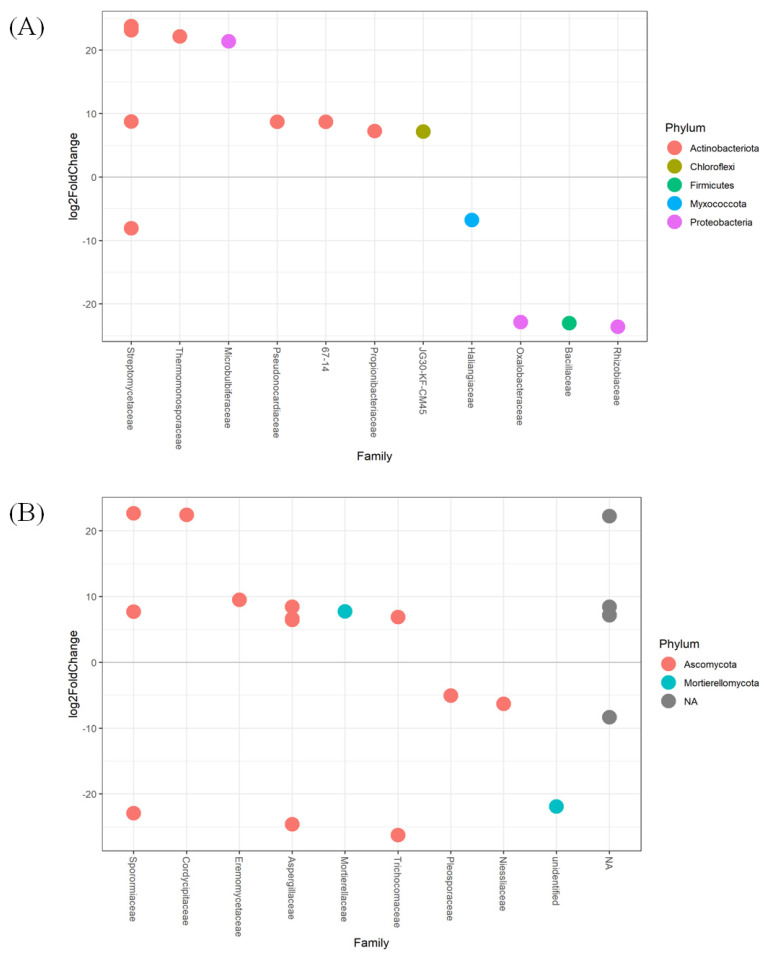
Bacterial (**A**) and fungal (**B**) amplicon sequence variants (ASVs) that significantly differ in abundance in unamended barley (control) vs. compost-amended barley when all barley growth stages are considered. Tillering (S1), booting (S2) and ripening stage (S3).

Differential abundance analysis identified amplicon sequence variants (ASVs) that were highly affected by the compost treatment compared to control soils sampled at the S1, S2 and S3 barley growth stages. Five bacterial phyla (14 ASVs) were enriched (Figure 6A) and they included members of *Actinobacteriota*, *Chloroflexi*, *Firmicutes*, *Myxococcota* and *Proteobacteria*. Most of the bacterial ASVs (nine ASVs) were more abundant in compost-treated soil than in the control. More specifically, bacterial families belonging to *Streptomycetaceae*, *Thermomonosporaceae*, *Microbulbiferaceae*, *Pseudonocardiaceae*, *67-14*, *Propionibacteriaceae* and *JG30-KF-CM45* dominated the compost-treated soil bacterial community, whereas families belonging to *Haliangiaceae*, *Oxalobacteraceae*, *Bacillaceae* and *Rhizobiaceae* were abundant in the control.

Regarding the fungal community, only two fungal groups (18 ASVs) were enriched (Figure 6B) and they included members of *Ascomycota* and *Mortierellomycota*. Most of the fungal ASVs (11 ASVs) were more abundant in compost-treated soil than in the control. More specifically, fungal families belonging to *Cordycipitaceae*, *Eremomycetaceae* and *Mortierellaceae* dominated the compost-treated soil fungal community, whereas families belonging to *Pleosporaceae*, *Niessliaceae* and Unidentified were abundant in the control. In contrast, fungal families belonging to *Sporomiaceae*, *Aspergillaceae*, *Trichomaceae* and NA dominated both treatments.

### 3.5. Metabolic Functional Features of the Microbial Community Present in Barley Rhizosphere

**Figure 7:**
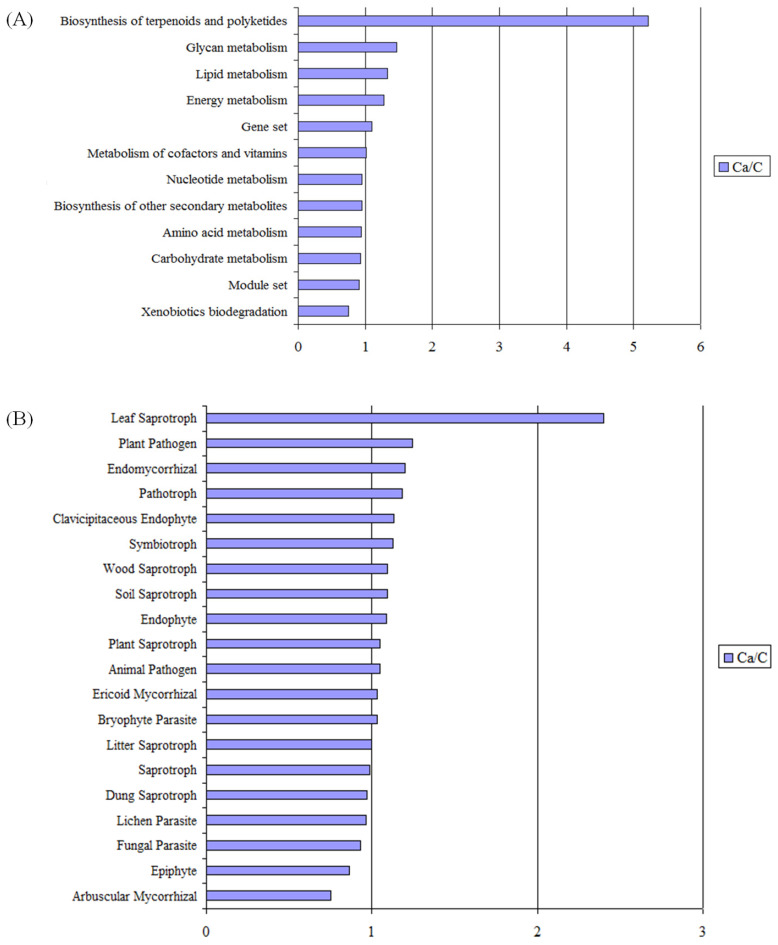
The bacterial (**A**) and fungal (**B**) functional features are based on the bacterial functions predicted by the KEGG module and the fungal functions potential estimated by the FUNGuild tool. The horizontal bars express the feature ratio value of the compost-amended soils (Ca) to unamended control soils (C).

In order to highlight the potential key roles of the microbial communities isolated from barley-cultivated soil amended or not with date palm waste compost, the PICRUSt2 (Phylogenetic Investigation of Communities by Reconstruction of Unobserved States) program was used to estimate the functional features of the bacterial communities according to the KEGG (Kyoto Encyclopedia of Genes and Genomes) module database (Figure 7A). Some metabolic functions such as energy metabolism, amino acid metabolism, metabolism of cofactors and vitamins, carbohydrate metabolism, biosynthesis of terpenoids and polyketides, gene set, lipid metabolism, xenobiotics biodegradation, biosynthesis of other secondary metabolites, glycan metabolism, nucleotide metabolism and module set were enriched in all the compost-treated soil groups. Compared with the control group, compost treatment significantly increased the relative abundance of biosynthesis of terpenoids and polyketides, glycan metabolism, lipid metabolism and energy metabolism. However, the relative abundance of xenobiotics biodegradation was significantly reduced. Furthermore, the relative abundance of most hypothetical bacterial functional taxa was not significantly affected by compost treatment.

FUNGuild tool was used to parse fungal taxonomy and establish ecological guilds (Figure 7B). Using the information from FUNGuild, twenty main ecological guilds such as wood saprotroph, animal pathogen, saprotroph, plant pathogen, pathotroph, plant saprotroph, symbiotroph, endophyte, soil saprotroph, dung saprotroph and epiphyte were found. Compared with the control group, the relative abundance of most hypothetical fungal functional taxa was not affected by the compost treatment. However, a significantly higher relative abundance of leaf saprotroph in compost-amended soil was revealed in comparison with the unamended control soils. All the results indicate that the most dominant functional guilds were symbiotroph and saprotroph, which may be favorable for barley plant growth promotion.

## 4. Discussion

The agricultural practices of applying biofertilizers and organic amendments such as compost increased diversity in the richness and structure of soil microorganisms, which play critical roles in soil quality, functions and plant productivity [33,34]. This study investigated the effects of compost application on the diversity, composition and functions of barley-cultivated soil bacterial and fungal communities using high-throughput sequencing (HTS) Illumina MiSeq technology based on amplicon sequencing of bacterial 16S rRNA gene and ITS rDNA region.

### 4.1. Application of Compost Affects Soil Microbial Community Abundance and Diversity

Using agricultural byproducts such as manure and compost may be an effective way to promote soil carbon sequestration and soil microorganism diversity and abundance, improving soil fertility and plant growth, which could lead to the sustainability of agricultural systems and food security [35]. Compost treatment significantly increased bacterial abundance, at tillering (S1) and booting (S2), and higher fungal abundance at the S1 and ripening (S3) barley growth stages compared to control treatment (Figure 1). These data suggested that compost application could promote soil bacterial and fungal growth. Moreover, these observations also implied that bacterial and fungal abundance were affected during the seasonal growth of barley. Previous studies reported that crop growth stages changed the soil bacterial and fungal abundance [36]. Similarly, Gao et al. [37] reported that application of organic amendment increased soil microbial abundance in wheat and soybean cropping systems. Similarly, the abundance of bacteria and fungi was significantly increased by the application of organic manure under jackfruit planting [38]. Likewise, Sharaf et al. [39] also suggested that bacterial abundance increased under compost amendment in the apple rhizosphere. Moreover, Liu et al. [12] reported that long-term application of biocompost increased soil fungal abundance. On the other hand, our findings are in discordance with the results by Azeem et al. [9], who found that amending turfgrass soils with biochar and compost did not affect bacterial 16S rRNA gene and fungal ITS rDNA region copy numbers.

It is generally recognized that the application of organic amendments alters the diversity (Shannon index), the richness (Chao1 index) and the composition of bacterial and fungal communities in soil [9]. Bacterial [40] and fungal [38] diversity commonly increased following application of organic fertilizers in comparison to unamended controls. These changes may be explained by either or both of the following factors: (1) an increase in the taxonomic spectrum of the bacterial and fungal community able to efficiently use new organic substrates, or (2) the introduction of bacterial or fungal species that inhabited the compost and were not native to treated-soil microbial communities. Our results showed that in comparison with the control, compost fertilization significantly increased the fungal richness and diversity indices at S1, while bacterial richness and diversity indices were significantly decreased at S1 and S3. In agreement with our findings, Liu et al. [12] and Azeem et al. [9] reported that compost application decreased soil bacterial diversity. Similarly, Gao et al. [37] and Luo et al. [41] found that the addition of organic fertilizers such as biochar increased soil fungal diversity. On the other hand, these results are not in line with the reports of Tao et al. [40] and Shen et al. [42], who showed that the application of biofertilizer significantly increased bacterial but decreased fungal diversity. One possible reason is that date palm waste compost stimulated the growth of some specific soil microorganisms and inhibited others. [12]. Another possible reason is that compost source materials and process, applied quantity, duration of experiment and soil properties affect soil bacterial community. In this study, short-term application of date palm waste compost may be the principal cause for the decrease in soil bacterial diversity. In addition, Gao et al. [37] reported that organic amendment (biochar) increased fungal richness and diversity compared with the control, which is consistent with our finding that compost application led to an increase in fungal richness and diversity in a barley field, especially at S1. The possible explanation for this result is date palm waste compost application could promote the soil fungal growth which was ascribed to change in soil pH, nutrient content and the increase in soil organic carbon (SOC) [43]. Interestingly, greater fungal diversity and abundance of bacterial and fungal communities could play an important role in the capacity of compost-treated soils to inhibit soilborne plant diseases, which may result in barley plant growth promotion and, thereby, in increased barley production under compost cropping practices [44].

The results of UniFrac-weighted principal coordinate analysis (PCoA) combined with a hierarchical clustering analysis showed that the control and compost treatment had been divided into two groups. Moreover, S1, S2 and S3 had a higher degree of similarity under compost treatment, while S1, S2 and S3 of the control treatment are in another group. These results demonstrated that the application of compost significantly affects soil bacterial and fungal community structure, especially observed at the S1 period. A similar result was reported by Liu et al. [12], that application of biocompost had a significant effect on alterations in the wheat and corn soil bacterial and fungal community structure. Overall, we can suggest that date palm waste compost application significantly affected soil microbial diversity.

### 4.2. Application of Compost Affects Bacterial and Fungal Community Composition

Previous research reported that organic fertilizers can cause changes in the taxonomic composition of soil microbial communities which play an important role in the N and P cycle and organic matter dynamics [45]. In this study, compost amendment affected the structure of soil microbial communities compared to control treatment. Moreover, microbial taxonomic composition varied between S1, S2 and S3 in compost-treated or untreated soils, suggesting that soil microbial communities respond differentially to compost amendment and barley crop growth, which will lead to changes in soil quality (Figure 3). Similar results were also found in previous studies suggesting that organic fertilizer application on soils and crop growth stages significantly affect the soil microbial composition [46].

In the present study, we found that *Proteobacteria*, *Actinobacteriota*, *Firmicutes*, *Chloroflexi* and *Gemmatimonadota* were the most abundant bacterial phyla identified in compost-treated soils at S1, S2 and S3, suggesting that these phyla may play an important role in the soil of the compost treatment. *Proteobacteria* relative abundance showed higher in compost-treated soils compared to untreated, and this phylum showed the highest proportion among bacterial phyla detected in the barley-cultivated 0–20 cm soil depth zone. These results generally agree with previous studies of Liu et al. [12], Su et al. [38] and Tao et al. [40], which reported that *Proteobacteria* was the most abundant phylum detected in soils under organic amendment application, suggesting that *Proteobacteria* relied more on labile C sources under nutrient input conditions [47]. In addition, the obtained results suggest an important role of *Proteobacteria* in the promotion of soil N transformation due to the significant correlation between most N metabolic pathways and *Proteobacteria* [12].

The phylum *Actinobacteriota* was also found in high abundance in this study. Indeed, *Actinobacteriota* showed significant responses to compost application at S1, S2 and S3, which is in line with several studies reporting that *Actinobacteriota* showed a positive response to organic substrate application, suggesting that this bacterial group is characteristic of nutrient-rich soil and high-carbon substrates [38]. Our observations support the work of Liu et al. [12], who found that the abundance of *Actinobacteriota* was increased by compost addition. This may be explained by the addition of compost that provides a carbon source for *Actinobacteriota* which participate in the degradation of organic matter. Interestingly, date palm waste compost increased the abundance of beneficial bacteria such as *Streptomyces* and *Nocardioides* within *Actinobacteriota* (Figure 4), which can produce antibiotics and consequently limit soilborne pathogens [48].

The phylum *Gemmatimonadota*, which is well known to have a broad spectrum of antagonistic activity against plant pathogenic fungi [49], was more abundant in compost-treated soil than in the control at S1, suggesting that compost application may be beneficial for alleviating the adverse effects of environmental stresses in barley fields.

The phylum *Firmicutes* was also found in high abundance in this study. Bacteria members of this phylum are oligotrophic and can resist extreme conditions and tolerate drought and heavy metals [50]. Moreover, previous study has shown that *Firmicutes* have beneficial bacteria associated with the C cycle [51]. In the current study, the phylum *Firmicutes* was greatly increased in control soil than in the compost-treated soil, especially at S1. These results lined up with the decreased abundance of plant-beneficial *Bacillus* within *Firmicutes* used as biocontrol agents and biofertilizers. This supports the work of Li et al. [52], who found that the addition of manure amendment largely decreased *Firmicutes* abundance while stimulating *Proteobacteria*. According to these authors, the possible explanation for this result is that the *Firmicutes* had lower metabolic potentials for utilizing manure-derived carbohydrates compared to *Proteobacteria*. Indeed, when the soils were amended with compost, the *Proteobacteria,* having such higher potential, outgrew the *Firmicutes* that initially dominated in control soils [52].

It is often reported that the *Chloroflexi* phylum is among the few taxa that show increased abundance in response to the addition of organic amendments such as biochar and compost [9]. The *Chloroflexi* phylum mostly contains anaerobes which can synthesize organic acids and hydrogen by fermentation of sugar and polysaccharides. In addition, this bacterial group accumulates the halogenated organic compounds produced by plant residue humification and enhanced soil C by CO_2_ fixing [53]. In this study, the relative abundance of *Chloroflexi* that increased under the compost fertilizer treatment, specifically at S1, compared to control soil might be a result of high organic matter substrate availability [54].

In this study, specifically, the relative abundance of *Verrucomicrobia* was observed in control soil at S1, suggesting that cropping systems and climate conditions affect *Verrucomicrobia* abundance. The phylum *Verrucomicrobia* was not revealed in compost treatment at S1, S2 and S3, suggesting that *Verrucomicrobia* communities decreased when soil fertility increased, supporting their important role in carbon cycling and carbohydrate degradation and that they could have a high affinity for nutrients such as total nitrogen content [55].

Various studies have shown that *Acidobacteriota* are the most abundant phylum and can promote the circulation of essential mineral nutrients in the soil, as well as decomposing complex carbohydrates such as polysaccharides, chitin, cellulose and lignin [56]. Moreover, *Acidobacteriota* can secrete exopolysaccharides (EPS) which promotes the absorption of water and nutrients by plants [57]. Interestingly, this bacterial group improves the formation of soil structure and aggregates and the accumulation of water and nutrients, which promotes plant–microbe interactions [58]. Kielak et al. [59] reported that *Acidobacteriota* directly promote plant growth by producing auxin (indole-3-acetic acid, IAA) and siderophore. In this study, we found that the relative abundance of *Acidobacteriota* was significantly increased in the compost treatment at S1 compared with the control, suggesting their great role in the promotion of nutrient enrichment and barley plant growth.

Similar to bacteria, date palm waste compost significantly altered fungal community structure (Figure 3B). A distinct difference in fungal community structure was revealed between compost-treated soils and untreated controls. Previous studies reported that fungal abundances at the order level were affected by compost treatment [12]. In the studied soils, *Eurotiales*, *Hypocreales*, *Sordariales* and *Pleosporales* dominated fungal orders (Figure 3B). Furthermore, *Ascomycota*, *Mortierellomycota* and *Basidiomycota* dominated fungal phyla (Figure 5). However, the relative abundance of these phyla exhibited different growth strategies with barley growth stages. These results support that *Ascomycota* was the most abundant phylum in compost-treated soil [12]. Furthermore, similar results have also been found in rhizospheric soil of *Coptis chinensis* Franch under a successive cropping system [60]. *Ascomycota,* which contained many beneficial and pathogenic fungi, was the most abundant phylum, suggesting the important role of compost to provide nutrients required for *Ascomycota* growth. Interestingly, date palm waste compost promoted the growth of beneficial fungi, such as *Chaetomium* within Ascomycota, and had inhibitory effects on plant pathogenic microorganisms such as *Alternaria*.

Under compost treatment, the relative abundance of *Humicola* significantly increased at S1, S2 and S3, while the relative abundance of *Chaetomium* significantly increased at S1, indicating compost promoted *Humicola* and *Chaetomium* population growth. *Humicola* are filamentous fungi found in the soil that are capable of producing enzymes that act synergistically for the degradation of lignocellulosic biomass which leads to the decomposition of residues [61]. *Chaetomium* are largely used to control and limit soilborne pathogens, thereby reducing pathogenic fungi and promoting barley plant growth [62]. Compost application significantly decreased the relative abundance of *Alternaria* at S1, suggesting that compost could be used as a biocontrol agent to control and limit soilborne pathogens. These results were in line with previous studies that indicated that application of organic amendments (compost) could suppress the abundance of soilborne pathogens [63]. Moreover, compost showed a significantly decreased relative abundance of *Aspergillus* at the ripening stage (S3), which can prevent barley seed rot [64].

The genus *Neocosmospora* are important plant pathogens, causing stem rot in many crops [65]. This fungus symptomatically causes stunted growth, yellowing of leaves and typical grayish-black streaking on plants. Compost application significantly decreased the relative abundance of *Neocosmospora* at S1, S2 and S3, suggesting that compost had significant effects on controlling soilborne diseases.

*Talaromyces* is a genus of fungi in the family *Trichocomaceae* which are proposed as major plant-growth-promoting fungi. According to Naragi et al. [66], this fungal group has a good phosphorus-dissolving effect and its application in soil promoted the growth of several plant species such as wheat, tomato and cucumber [67]. Compost significantly increased the abundance of *Talaromyces* at S1 and S2, which could promote the growth of barley during the vegetative growth stages. In this context, *Mortierella* plays an important role in organic carbon decomposition, nutrient uptake efficiency and crop protection against adverse conditions [68]. Compost treatment significantly increased the abundance of *Mortierellomycota* and *Mortierella* at the tillering stage, suggesting a possible critical role of these beneficial microorganisms in barley growth promotion. However, the abundance of *Mortierella* decreased at the ripening stage, which may be explained by their negative correlation with soil nutrients [12].

In the fungi community, the relative abundance of *Basidiomycota* decreased by application of compost at S1 and S2, which is consistent with Sun et al. [69] and Jones et al. [70], who reported that this decrease in the relative abundance of *Basidiomycota* could be attributed to the rapidity of metabolization of organic matter in the topsoil by *Basidiomycota*.

It is well reported that *Penicillium* spp. may promote plant growth by synthesizing plant hormones, promoting solubilization of phosphate, production of antibiotics and protection from abiotic stresses such as salinity and the induction of plant resistance [71,72]. Compost treatment increased the relative abundance of *Penicillium* at S1. Similar results were reported by Mącik et al. [73], who showed that biofertilizer treatment increased *Penicillium* abundance.

### 4.3. Effect of Date Palm Waste Compost Application on the Overall Function of Soil Microbial Community

PICRUSt and FUNGuild are useful databases for functional analysis of soil bacteria and fungi, respectively. The application of compost changed the overall functions of soil bacterial and fungal communities resulting in differences among the functional diversity of soil microorganisms between individual treatments. The PICRUSt results showed that energy metabolism, amino acid metabolism, metabolism of cofactors and vitamins and carbohydrate metabolism were the most important of the 12 categories of metabolic pathways, which were similar to results reported by Liu et al. [12]. It is well known that energy metabolism, amino acid metabolism and carbohydrate metabolism are associated to the main functions of bacteria [74]. These observations suggest that application of compost led to increased soil nutrients such as organic carbon, increased metabolic substrate availability for bacteria and, thereby, resulted in changes in metabolic functions of bacterial communities.

The high proportion of certain metabolic pathways such as carbohydrate and amino acid suggested that adding compost to the soil may increase the metabolic properties of bacteria towards certain compounds, especially related to the C, N and P cycles, and consequently shift the metabolic function of bacteria. Similarly, Sharaf et al. [75] and Liu et al. [12] found that C, N and P cycling were the most prominent functional change associated with the soil bacterial community under compost application. Moreover, this study’s results are in accordance with previous research showing that application of bio-organic fertilizer improved the N and C cycling processes in tea plantation soils [9]. The abundance of energy metabolism, amino acid metabolism and carbohydrate metabolism in compost-treated soils compared to controls suggests a more eutrophic state of compost-treated soils compared to the controls [76].

Through FUNGuild analysis, fungal communities were classified into 11 trophic modes, which is in agreement with Alami et al. [60]. The saprotroph dominated the functional guilds in compost-treated soils, which may play critical roles in organic decomposition, nutrient cycle and barley plant growth promotion and resulted in improving barley seed quality [32]. Based on fungal community composition, the studied soil fungal communities are dominated by *Ascomycota* and *Basidiomycota*, which employ saprophytic or symbiotic nutritional approaches and play important roles as decomposers in the soil and in barley crop health and nutrition [77]. Here, plant pathogens abundance was lower than animal pathogens, which is consistent with Alami et al. [60].

## 5. Conclusions

In summary, this study demonstrates that the application of date palm waste compost significantly increased soil microbial richness and diversity at the tillering, booting and ripening stages of barley growth. Furthermore, the amendment of compost significantly influenced the composition and structure of soil microbial communities. Compost treatment enriched the beneficial microorganisms (such as *Chaetomium*, *Actinobacteriota*, *Talaromyces* and *Mortierella)* and reduced the harmful microorganisms (such as *Alternaria*, *Aspergillus* and *Neocosmospora*). In addition, the application of compost altered the soil bacterial and fungal community functions, their metabolic functions and trophic modes. Overall, this study’s results indicated that compost can promote beneficial microorganisms which leads to increased availability of essential nutrients to barley plants, producing substances involved in barley plant growth and suppressing pathogens, thereby improving the productivity and disease resistance of barley crops. Further studies are needed to focus on the long-term impacts of date palm waste compost on the microbial community showing specific soil functions in the barley rhizosphere.

## Figures and Tables

**Table 1:** Soil physiochemical properties.

Parameters	Value
Clay (%)	5.50
Silt (%)	8.30
Sand (%)	84.40
Soil texture	Sandy
pH	7.50
EC (dS m^−1^)	4.02
Organic matter (%)	0.91
Total organic carbon (%)	0.53
Total N (mg kg^−1^ soil)	280
Available P (mg kg^−1^ soil)	4.92
Exchange K (mg kg^−1^ soil)	292
Total coliforms (MPN g DW^−1^ soil)	24 × 10^2^
Fecal coliforms (MPN g DW^−1^ soil)	<0.3
Escherichia coli (MPN g DW^−1^ soil)	<0.3
Fecal Streptococci (MPN g DW^−1^ soil)	39 × 10^2^

**Table 2:** Characteristics of date palm waste compost.

Parameters	Value
Total organic carbon (%)	18.58
Total N (%)	1.21
C/N	15.36
P (%)	0.54
K (%)	0.95
Ca (%)	8.18
Mg (%)	1.05
Na (%)	0.42
Alkalinity (% CaCO_3_)	11.50
Zn (mg kg^−1^ DW compost)	70.10
Fe (g kg^−1^ DW compost)	70
Mn (mg kg^−1^ DW compost)	130
Cu (mg kg^−1^ DW compost)	11.60
Cd (mg kg^−1^ DW compost)	0.20
Pb (mg kg^−1^ DW compost)	4.15
Cr (mg kg^−1^ DW compost)	11.50
Ni (mg kg^−1^ DW compost)	5.88
Total coliforms (MPN g^−1^ DW compost)	143.33 ± 5.77
Fecal coliforms (MPN g^−1^ DW compost)	120 ± 17.32
Escherichia coli (MPN g^−1^ DW compost)	114 ± 23.79
Fecal Streptococci (MPN g^−1^ DW compost)	114.33 ± 23.80
Salmonella spp. (MPN g^−1^ DW compost)	<0.3
Shigella spp. (MPN g^−1^ DW compost)	<0.3

## Data Availability

Data are contained within the article.
